# Supplementary material for: Stress-induced brain responses are associated with BMI in women
Source: Commun Biol. 2023 Oct 11;6:1031. doi: 10.1038/s42003-023-05396-8 (PMC10567923; doi:10.1038/s42003-023-05396-8)
Supplement: Supplementary file 2 — Supporting Information [file 42003_2023_5396_MOESM2_ESM.pdf]

Supplementary Information:

Stress-induced brain responses are associated with BMI in women

Anne Kühnel[1,2,3*], Jonas Hagenberg, Janine Arloth, Maik Ködel, Michael Czisch[4], Philipp G. Sämann[4], BeCOME working group, Elisabeth B. Binder[1*], Nils B. Kroemer[1,5]

[1] Department of Psychiatry and Psychotherapy, University of Bonn, Bonn, Germany

[2] Department of Translational Research in Psychiatry, Max Planck Institute of Psychiatry, Munich, Germany

[3] International Max Planck Research School for Translational Psychiatry (IMPRS-TP), Munich, Germany

[4] Max Planck Institute of Psychiatry, Munich, Germany

[5] Department of Psychiatry and Psychotherapy, Tübingen Center for Mental Health (TüCMH), University of Tübingen, Tübingen, Germany

Corresponding authors*

A. Kühnel, anne_kuehnel@psych.mpg.de

E. Binder, binder@psych.mpg.de

Post-hoc Analysis of regions of interest Posterior Insula/Midbrain:

To better characterize the associations of BMI and stress-induced activation, we extracted average beta values from ROIs [9] containing the significant clusters. We then performed post-hoc regression analyses on the whole sample including a Sex*BMI interaction as well as separately for males and females to explore and describe potential sex effects. The association with BMI was only significant in females (substantia nigra (SN): b = -0.05, p = .004, posterior insula R: b = -0.06 p < .001, posterior insula L: b = -0.03, p = .008) but not males (SN: b = -0.001, p = .95, posterior insula L: b = -0.03, p = .069, posterior insula R: b = 0.005, p = .75, Figure 3B).

Figures

Figure S1: Detailed description of the psychosocial stress task[5]. Before the stress phase, participants were informed about being recorded in the following trials. Additional aversive verbal feedback (verbal FB) about unsatisfactory performance was given in the 2nd and 4th rest period of the *Stress* condition. Saliva sampling was done in all participants (N=192) and in subsample of n=73 participants blood samples were taken to assess the cortisol response with higher temporal resolution.

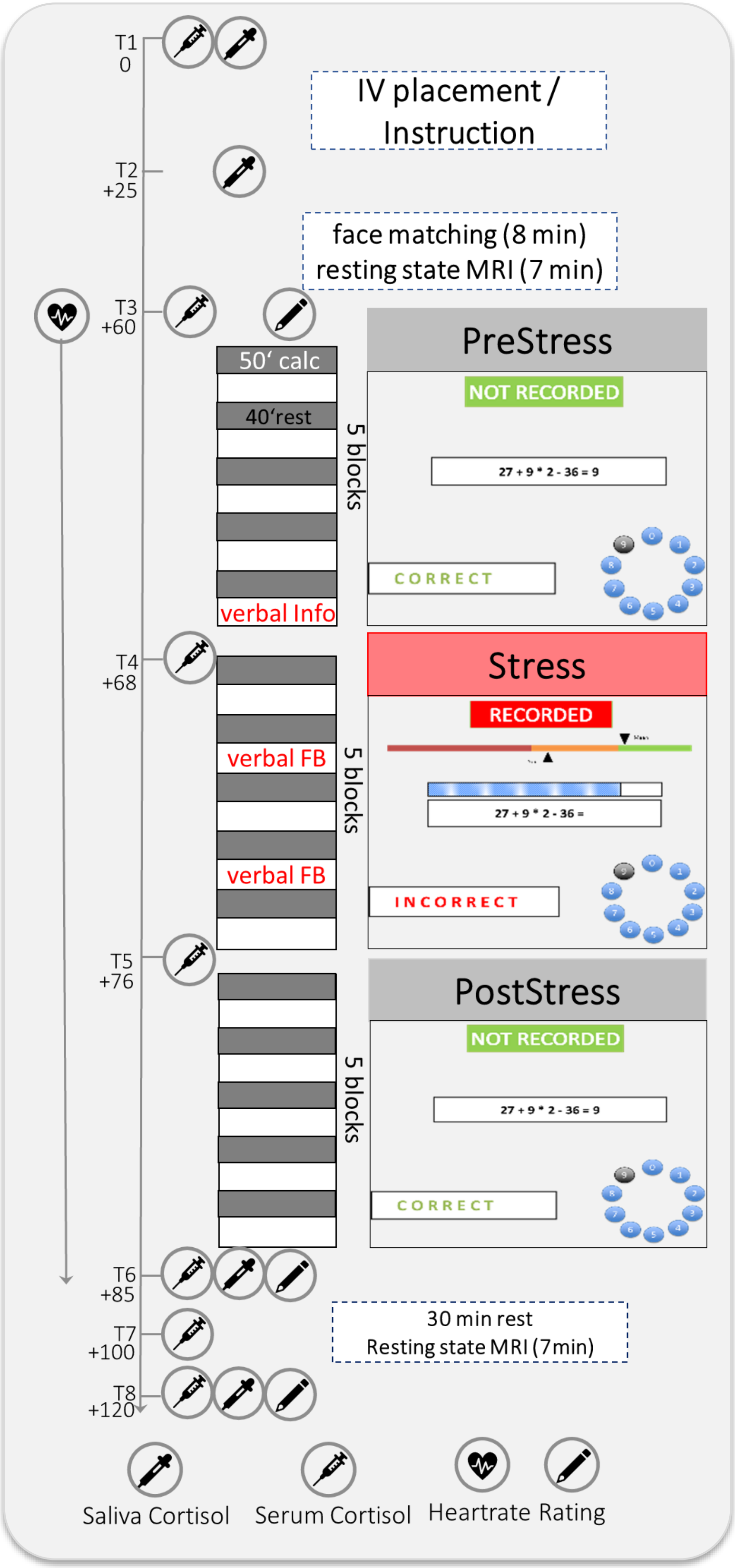

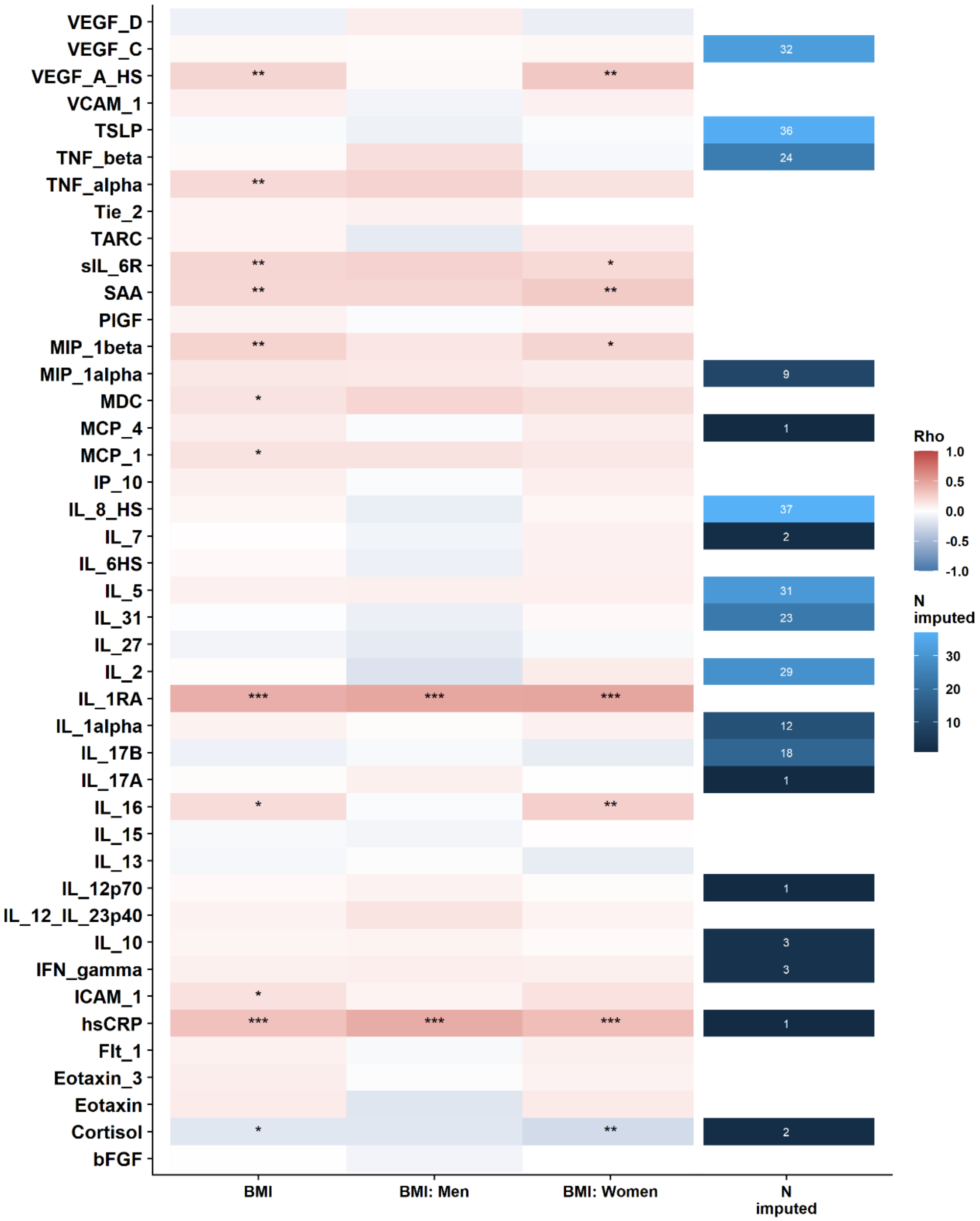

Figure S2: Uncorrected, partial correlations of body mass index (BMI) with all different immune markers, for the complete sample and men and women separately in a larger sample (N=198). All correlations are corrected for age, diagnosis, and current psychiatric medication. The last column shows the number of values that have been imputed. White indicates no values had to be imputed.

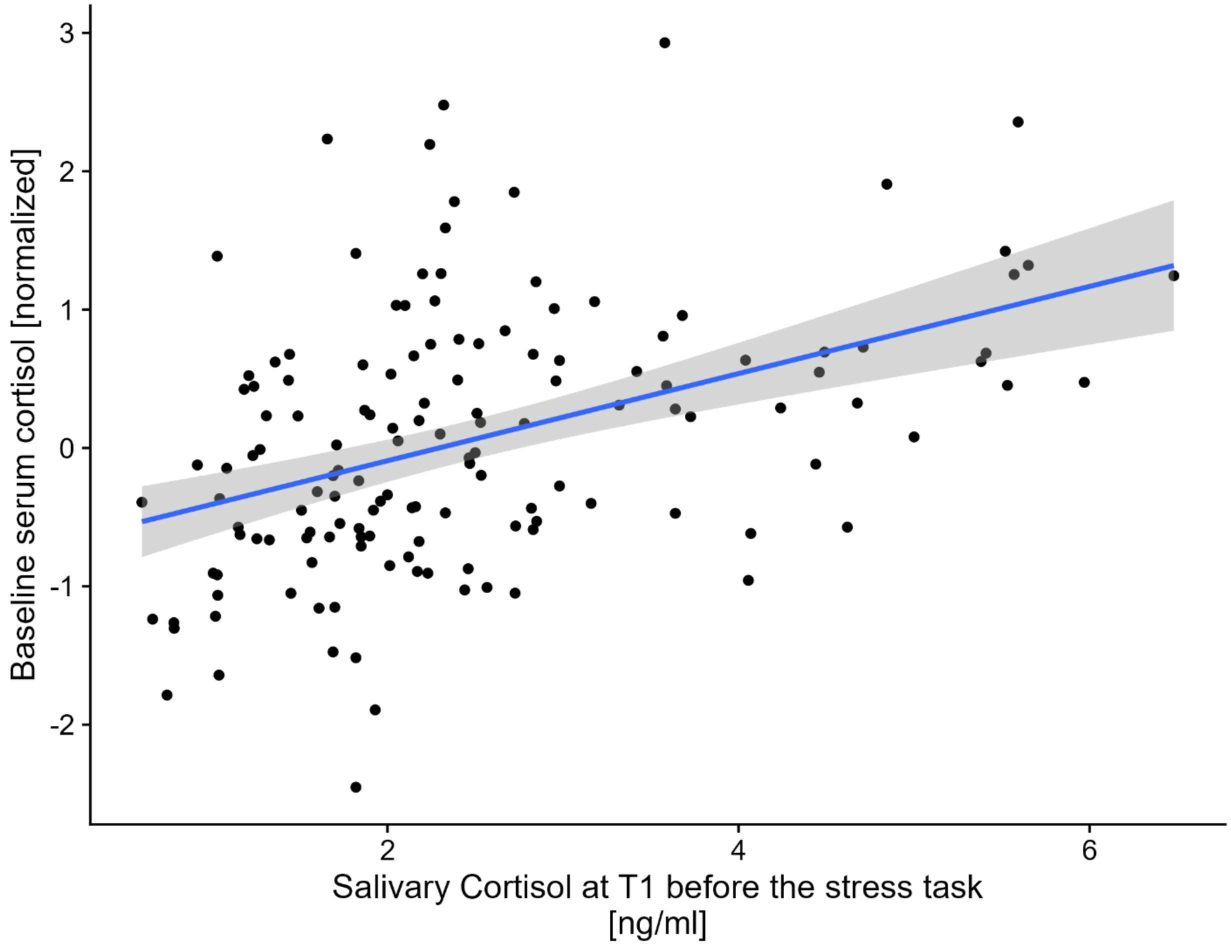

Figure S3: Correlation ($r(140) = .42$, $p < .001$) of the baseline morning cortisol assessment (measured from a serum sample together with the cytokines) at separate day and the first salivary cortisol sample before the stress task in n=142 participants. This sample was taken at approximately 10am and after participants had already completed a fear extinction paradigm.

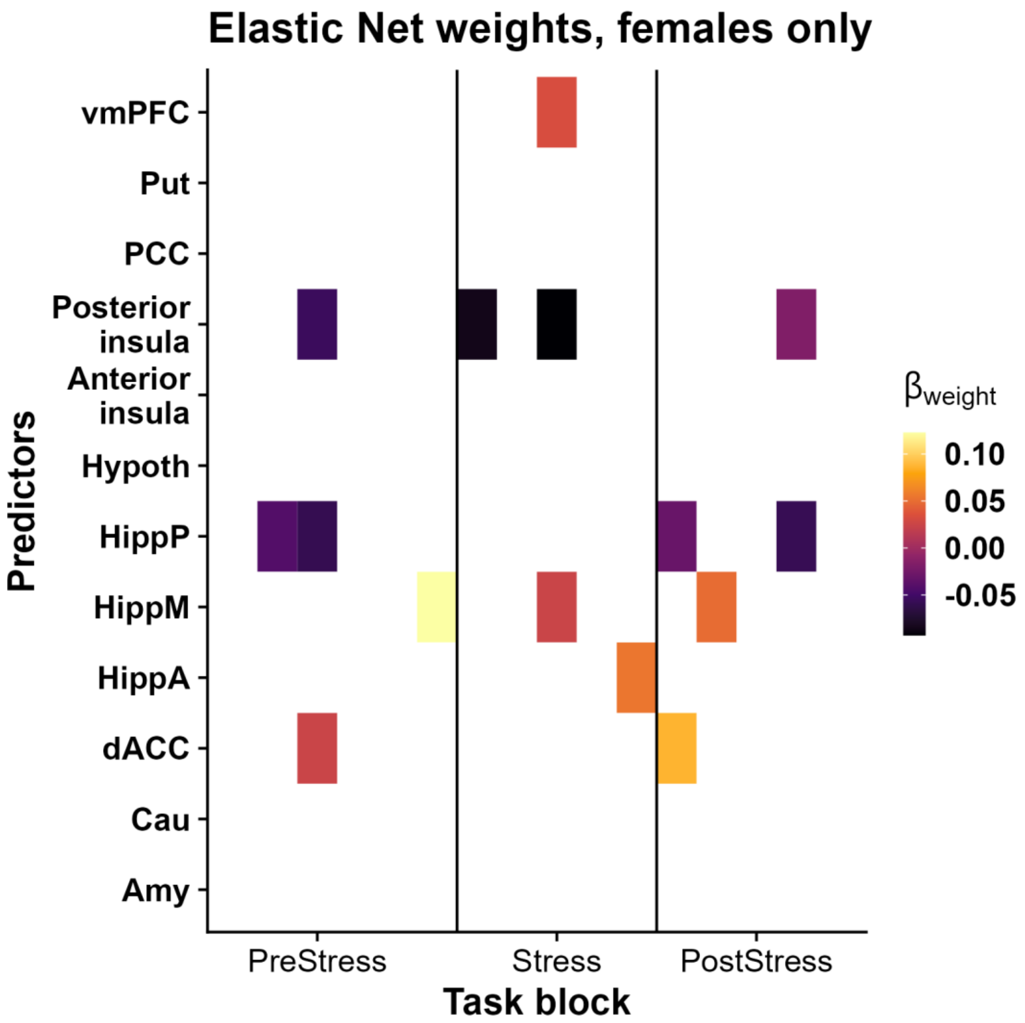

Figure S4: Weights of the elastic net model retrained in the female sample only (n=120). In comparison to the combined male + female model, activation from more timepoints of the hippocampus and posterior insula contribute to a successful prediction. Additionally, there are also contributions of the dorsal anterior cingulate cortex and ventromedial prefrontal cortex. vmPFC = ventromedial prefrontal cortex, Put = Putamen, PCC = posterior cingulate cortex, Hypoth = Hypothalamus, HippP = posterior hippocampus, HippM = medial hippocampus, HippA = anterior hippocampus, dACC = dorsal anterior cingulate cortex, Cau = caudate, Amy = amygdala.

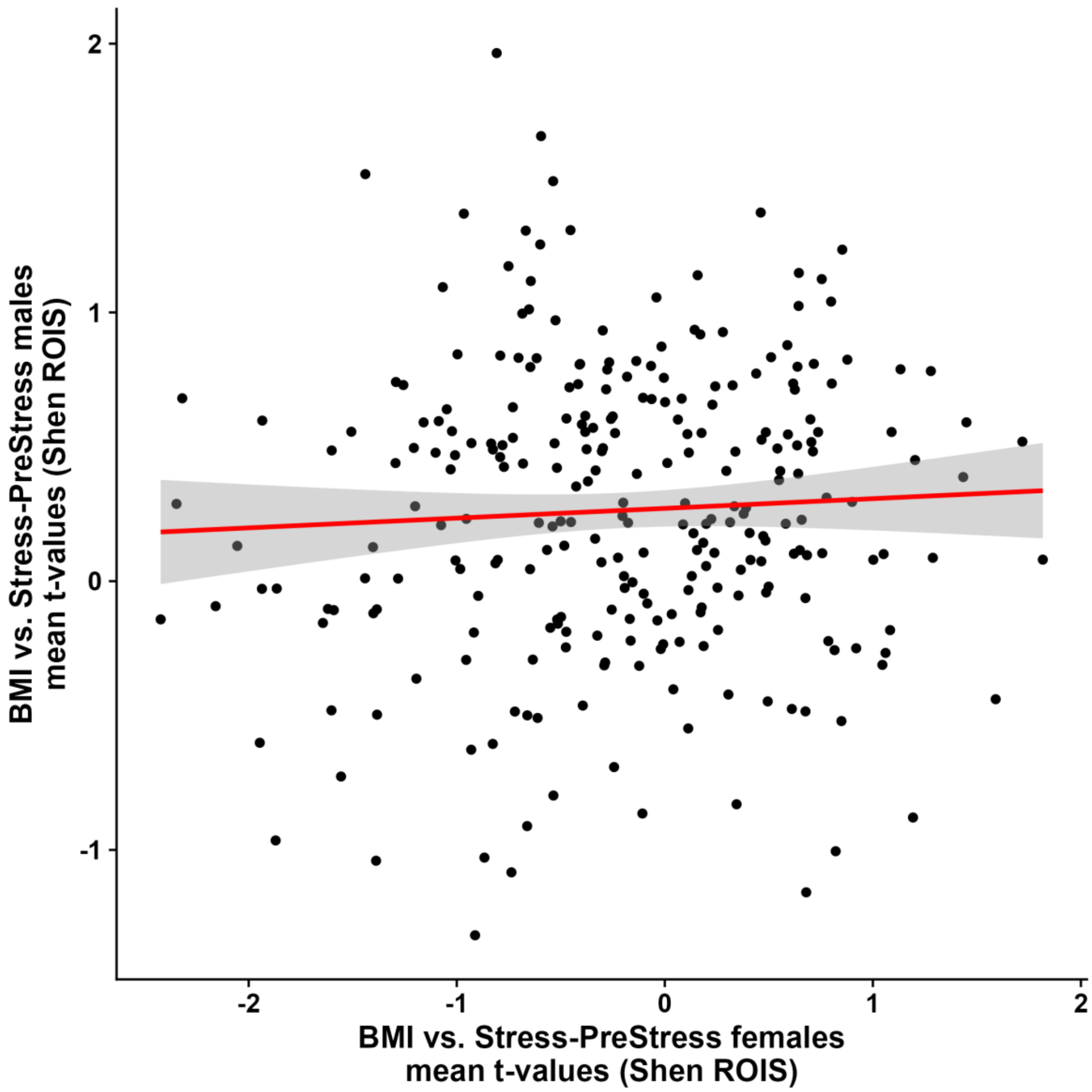

Figure S5: Activation patterns correlated with BMI in males and females are not associated. Avergage stress-induced (Stress – PreStress) activation associated with from all n=268 regions of interest in the Shen Atlas is not correlated between males and females (r=.05, p=.38)

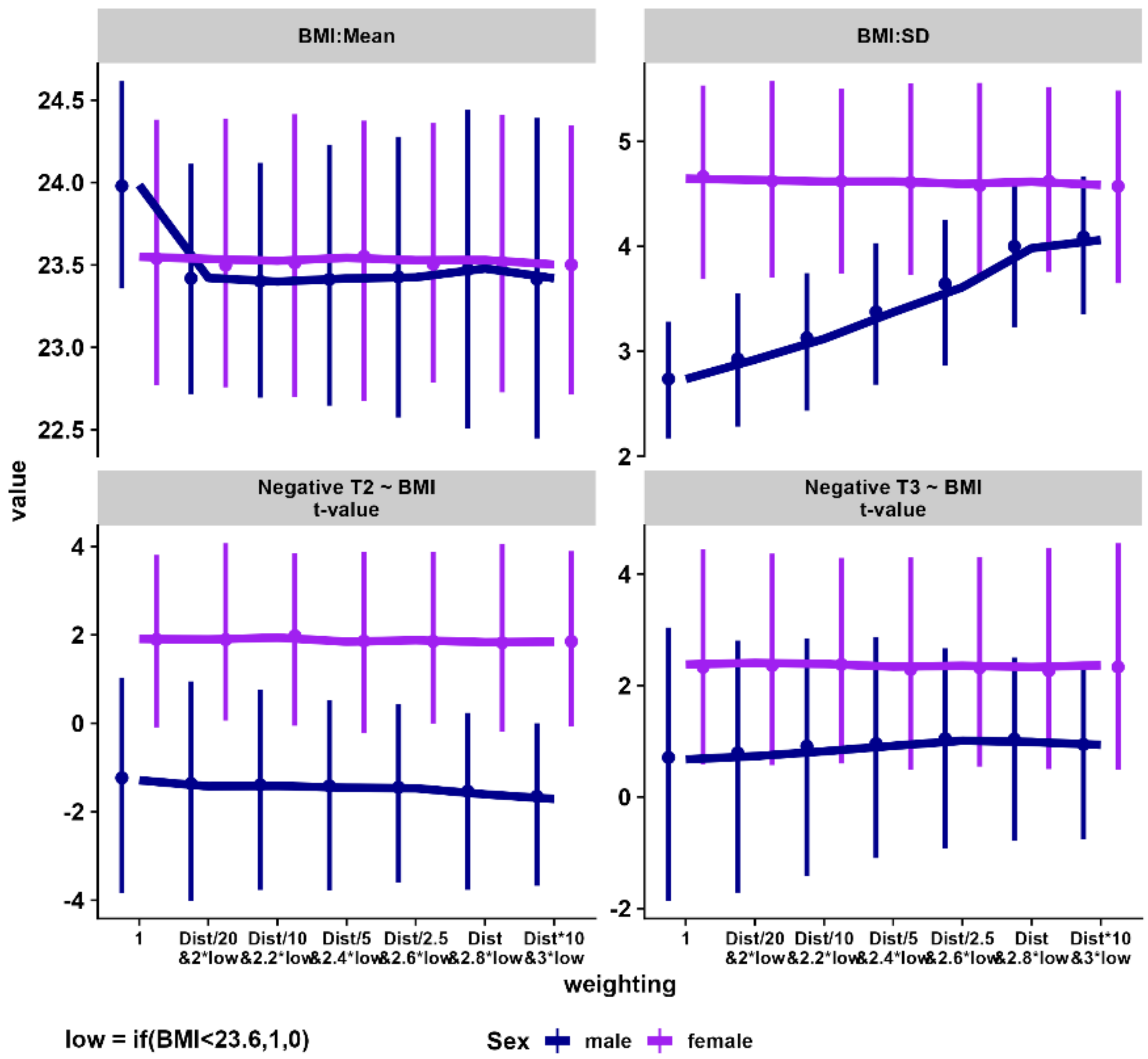

Figure S6: Bootstrapped associations of BMI with negative affect after the task. In the analysis, data was resampled in males so that the mean (upper right) and standard deviation (upper left) approach the female distribution. For each weighting scheme (x-axis), data was resampled 1,000 times to derive average estimates and 95% confidence intervals. Associations of BMI with stress-induced negative affect in males did only change marginally after adjusting the weights.

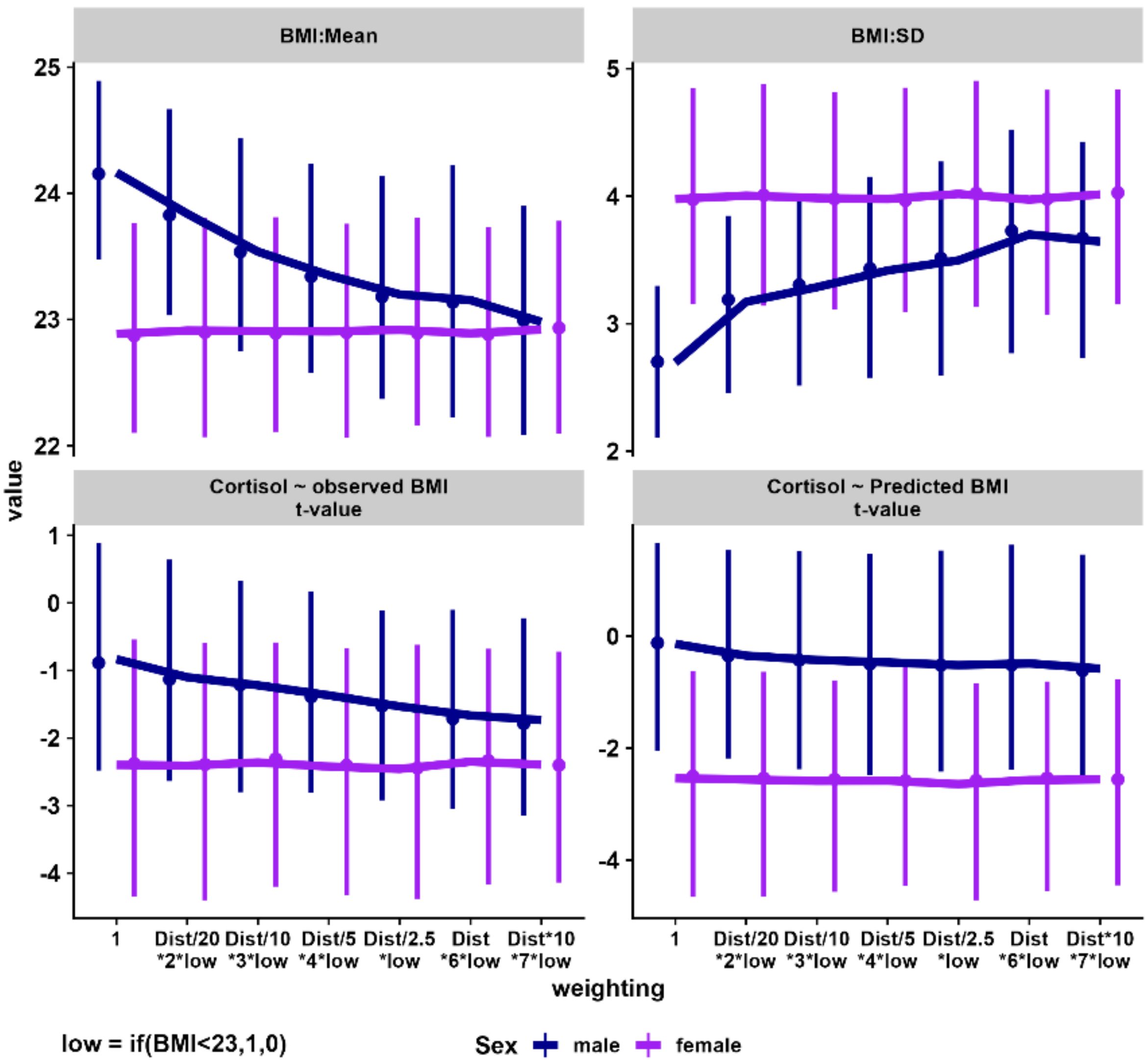

Figure S7: Bootstrapped associations of negative affect after the task with BMI. In the analysis, data was resampled in males so that the mean (upper left) and standard deviation (upper right) gradually approach the female distribution. For each weighting scheme (x-axis), data was resampled 1,000 times to derive average estimates and 95% confidence intervals. Associations of the observed BMI with baseline cortisol became increasingly similar between males and females if the distributions became more similar. In contrast, this was not seen for the correlation with the predicted

Tables

Table S1: Current and lifetime prevalence of psychiatric disorders identified using the CIDI in the present sample.

	**12-months diagnosis N(%)**	**Lifetime diagnosis N(%)**
Substance use disorders (F1)	8 (4%)	42 (22%)
Mood disorders (F3)	65 (35%)	75 (39%)
Anxiety-related disorders (F4)	101 (53%)	122 (64%)
Other disorders	8 (4%)	14 (7%)
No diagnoses	74 (39%)	50 (26%)
1 diagnosis	56 (29%)	54 (28%)
2 diagnoses	51 (27%)	59 (31%)
3 and more diagnoses	9 (5%)	27 (14%)

Note: Anxiety disorders (F4) include specific phobias. Stress-related disorders in the last 12 months are defined as participants with a mood or anxiety-related disorder within the last 12 months excluding specific phobias. The control includes all other participants that might still receive diagnoses from other axes (e.g. substance use disorder: smoking).

Table S2: Number of participants included across all analyses.

N	Subjective	Endocrine	Heart rate	Neural	Immune markers
BMI	189	186	165	190	148

Note: Exclusion reasons: Endocrine (salivary cortisol) not enough material, Heart rate insufficient data quality

Table S3: No sex-specific effects of age on BMI

Dependend variable	Predictors	Estimate	Std. error	p-value
BMI	Sex	-0.22	0.29	0.44
	Age	0.64	0.29	0.028
	Sex* Age	-0.04	0.30	0.89

Table S4: Multiple regression models predicting stress responses (subjective, cardiovascular, endocrine) by sex, BMI, and their interaction.

Group	Characteristic	Beta	95% CI[1]	p-value
ΔHR PostStress	Sex	-0.39	-2.0, 1.2	0.64
	BMI	-0.61	-1.6, 0.35	0.21
	BMI * Sex	-0.66	-2.9, 1.5	0.56
ΔHR Stress	Sex	-0.38	-2.5, 1.8	0.73
	BMI	-1.0	-2.2, 0.28	0.13
	BMI * Sex	-1.4	-4.3, 1.5	0.35
ΔCortisol T6	BMI	0.19	-0.17, 0.55	0.30
	Sex	0.37	-0.25, 1.0	0.24
	BMI * Sex	0.44	-0.42, 1.3	0.32
ΔCortisol T8	BMI	0.09	-0.16, 0.33	0.49
	Sex	-0.10	-0.51, 0.32	0.65
	BMI * Sex	0.35	-0.23, 0.93	0.24
ΔNegative affect T6	**BMI**	**1.5**	**0.01, 2.9**	**0.047**
	Sex	-1.8	-4.3, 0.66	0.15
	BMI * Sex	-2.9	-6.3, 0.55	0.10
ΔNegative affect T8	**BMI**	**1.2**	**0.20, 2.2**	**0.019**
	Sex	-0.50	-2.2, 1.2	0.56
	BMI * Sex	-0.50	-2.9, 1.9	0.67
ΔPositive affect T6	BMI	0.09	-0.60, 0.78	0.79
	Sex	0.23	-0.94, 1.4	0.70
	BMI * Sex	0.52	-1.1, 2.2	0.54
ΔPositive affect T8	BMI	-0.52	-1.2, 0.15	0.12
	Sex	0.01	-1.1, 1.1	0.98
	BMI * Sex	1.0	-0.58, 2.6	0.21

[1]CI = Confidence Interval, BMI = Body mass index, In the multiple regression sex was dummy-coded with 0 = females and 1 = males. All models additionally included age, diagnosis status, cortisol response to the placement of an IV (responder = 1, non-responder 0) and medication status.

Supplementary References

1. Janke, W. Befindlichkeitsskalierung durch Kategorien und Eigenschaftswörter: BSKE (EWL) nach Janke, Debus, Erdmann und Hüppe. *Test und Handanweisung. Unveröffentlichter Institutsbericht, Lehrstuhl für Biologische und Klinische Psychologie der Universität Würzburg* (1994).
2. Janke, W. & Debus, G. *Die Eigenschaftswörterliste: EWL*. (Verlag für Psychologie CJ Hogrefe, 1978).
3. Elbau, I. G. *et al.* The brain's hemodynamic response function rapidly changes under acute psychosocial stress in association with genetic and endocrine stress response markers. *PNAS* 201804340 (2018) doi:10.1073/pnas.1804340115.
4. Ising, M. *et al.* Polymorphisms in the FKBP5 gene region modulate recovery from psychosocial stress in healthy controls. *European Journal of Neuroscience* **28**, 389–398 (2008).
5. Kühnel, A. *et al.* Psychosocial stress reactivity habituates following acute physiological stress. *Human Brain Mapping* **41**, 4010–4023 (2020).
6. Kuhn, L. *et al.* The association of the 5-HTTLPR polymorphism and the response to different stressors in healthy males. *J Neural Transm* **128**, 1347–1359 (2021).
7. Shilton, A. L., Laycock, R. & Crewther, S. G. The Maastricht Acute Stress Test (MAST): Physiological and Subjective Responses in Anticipation, and Post-stress. *Frontiers in Psychology* **8**, 567 (2017).
8. Vest, A. N. *et al.* An open source benchmarked toolbox for cardiovascular waveform and interval analysis. *Physiol. Meas.* **39**, 105004 (2018).
9. Shen, X., Tokoglu, F., Papademetris, X. & Constable, R. T. Groupwise whole-brain parcellation from resting-state fMRI data for network node identification. *Neuroimage* **0**, 403–415 (2013).
