# Supplementary material for: Stress-induced brain responses are associated with BMI in women
Source: Commun Biol. 2023 Oct 11;6:1031. doi: 10.1038/s42003-023-05396-8 (PMC10567923; doi:10.1038/s42003-023-05396-8)
Supplement: Supplementary file 3 — Description of Additional Supplementary Files [file 42003_2023_5396_MOESM3_ESM.pdf]

Description of Additional Supplementary Files

File name: Supplementary Data 1

Description: Contains source data for all figures (main text and supplement) within the paper.
